# Gastrolaryn^®^ Stick Following Proton Pump Inhibitor Treatment in Patients with Gastroesophageal Reflux Disease: A Multicenter Real-World Observational Survey

**DOI:** 10.3390/jcm15218516

**Published:** 2026-11-02

**Authors:** Giorgio Ciprandi, Ignazio La Mantia, Attilio Varricchio

**Affiliations:** 1 Medicine and Healthcare Science Department, University of Molise, 86100 Campobasso, Italy; 2 Department of Otorhinolaryngology, University Kore, 94100 Enna, Italy

**Keywords:** gastroesophageal reflux disease, GERD, NERD, sodium alginate, Gastrolaryn^®^ Stick, GERD Impact Scale, GIS, real-world study, maintenance therapy, proton pump inhibitors

## Abstract

**Background:** Symptom management after proton pump inhibitor (PPI) treatment remains an important issue in gastroesophageal reflux disease (GERD). Objective: To describe symptom distributions during a sequential PPI and Gastrolaryn^®^ Stick management pathway in routine outpatient practice. **Methods:** In a multicenter, prospective observational survey, 263 GERD patient records were collected at baseline (T0) by 17 gastroenterologists. The intended management sequence comprised 8 weeks of esomeprazole 40 mg daily (T0–T1) followed by 8 weeks of Gastrolaryn^®^ Stick (one stick twice daily; T1–T2). Outcomes included GERD Impact Scale (GIS) response distributions and routine-care characteristics. Analyses were descriptive and based on available records at each survey timepoint. **Results:** Records were available for 124 patients at T1 (47.1% of baseline) and 112 at T2 (42.6%). Across recorded GIS item responses, the proportion rated “every day” was 12.8%, 0.9%, and 0.3% at T0, T1, and T2, respectively; the corresponding “never” proportions were 24.8%, 44.5%, and 58.4%. These are unpaired, timepoint-specific descriptive summaries and do not establish individual improvement. Investigators submitting records declined from 17 to 11 and 7. **Conclusions:** Among available records, symptom-frequency distributions were more favorable at later survey timepoints. Substantial missing follow-up, changing site participation, the lack of a comparator and unavailable patient-level paired analyses prevent causal attribution, assessment of relapse prevention, or definitive conclusions about maintenance effectiveness. The survey nevertheless documents management and follow-up patterns encountered in routine practice.

## 1. Introduction

Gastroesophageal reflux disease (GERD) is one of the most prevalent chronic gastrointestinal conditions in Western countries, with an estimated prevalence of 10–20% of the adult population in Europe and North America [1,2]. According to the Montreal Consensus definition, GERD is “a condition that develops when the reflux of stomach contents causes troublesome symptoms or complications” [3]. Moreover, recent updates in gastrointestinal consensus guidelines underscore a shift toward a multi-parametric diagnostic approach for esophageal symptoms. Specifically, the Lyon Consensus 2.0 refines the objective physiological criteria required for a conclusive diagnosis of actionable GERD [4]. The Rome V framework separately addresses disorders of gut–brain interaction, which may enter the differential diagnosis of reflux-like symptoms [5]. The disease encompasses a broad clinical spectrum, ranging from non-erosive reflux disease (NERD), the most common phenotype, accounting for up to 70% of cases, to erosive esophagitis, Barrett’s esophagus, and esophageal adenocarcinoma [6,7]. Typical symptoms include heartburn and regurgitation, while atypical manifestations such as chest pain, chronic cough, and epigastric discomfort are also frequently reported [3]. GERD significantly impairs health-related quality of life, affecting sleep, work productivity, dietary habits, and social functioning [8,9].

The pathophysiology of GERD is multifactorial. The primary mechanism involves transient lower esophageal sphincter relaxations (TLOSRs), which allow gastric contents, including hydrochloric acid, pepsin, and bile salts, to reflux into the esophagus [10]. Contributing factors include reduced esophageal clearance, impaired mucosal defence mechanisms, delayed gastric emptying, hiatal hernia, and increased intra-abdominal pressure [10,11]. Lifestyle factors such as obesity, dietary habits (high-fat meals, coffee, alcohol, carbonated beverages), smoking, and supine postprandial positioning are well-established risk factors that amplify reflux episodes and symptom severity [12,13]. The high prevalence of these modifiable risk factors in the general population underscores the importance of lifestyle modification as a cornerstone of GERD management, alongside pharmacological therapy. In this regard, proton pump inhibitors (PPIs) represent the established first-line pharmacological treatment for GERD, providing effective acid suppression and symptom relief in the majority of patients [14,15,16]. However, PPIs are not without limitations. A significant proportion of patients, estimated at 10–40%, experience incomplete symptom control or symptom relapse after PPI discontinuation, which may include, but are not synonymous with, rebound acid hypersecretion [17,18]. In addition, long-term PPI use is associated with potential adverse effects including hypomagnesemia, vitamin B12 deficiency, increased risk of enteric infections, and bone fractures [17,18]. These concerns have driven increasing interest in non-pharmacological and complementary approaches to GERD management, particularly for maintenance therapy following an initial PPI course [19]. As a result, among the alternative and complementary therapeutic options, alginate-based formulations have attracted considerable attention. Sodium alginate is a naturally occurring polysaccharide derived from brown seaweed (Phaeophyceae), composed of linear chains of β-D-mannuronic acid and α-L-guluronic acid residues [20]. When ingested, sodium alginate reacts rapidly with gastric hydrochloric acid to form a viscous gel of alginic acid, which floats on the surface of the gastric contents—a phenomenon known as the “raft mechanism” [21]. This gel raft acts as a physical barrier at the gastro-oesophageal junction, preventing the reflux of acidic gastric contents into the esophagus without systemic acid suppression [22]. Trials and meta-analyses suggest that selected alginate-based formulations can alleviate GERD symptoms, although comparisons with PPIs and benefits of combination therapy vary by formulation and study [23,24,25]. In this regard, Gastrolaryn^®^ Stick (NÓOS S.r.l., Rome, Italy) is a multi-component food supplement specifically formulated to manage gastric reflux. Its composition combines the mechanical anti-reflux properties of sodium alginate with the mucosal protective, anti-inflammatory, and soothing properties of additional active components. In particular, (i) sodium alginate, as described above, forms a gel raft with a near-neutral pH, minimizing esophageal mucosal irritation [21,22]; (ii) hyaluronic acid (sodium hyaluronate) is a high-molecular-weight glycosaminoglycan ubiquitously present in connective tissues and mucosal surfaces [26,27]. In the gastrointestinal tract, hyaluronic acid exerts cytoprotective effects by forming a viscoelastic hydration layer over the esophageal and gastric mucosa, promoting epithelial repair and reducing inflammation-mediated mucosal damage [27]; and (iii) *Althaea officinalis* L. root dry extract (marshmallow root) has been used in traditional medicine for centuries as a demulcent and emollient agent. Its pharmacological activity is attributed primarily to its high mucilage content (polysaccharides including arabinogalactans and glucuronoxylans), which forms a protective coating over irritated mucosal surfaces, reducing friction, inflammation, and pain [28]. Preclinical studies have confirmed its anti-inflammatory and mucosal-protective properties in gastrointestinal models [29]; (iv) *Musa paradisiaca* L. fruit powder, unripe banana powder has a well-documented role in gastric mucosal protection [30]. Its active constituents include leucocyanidin (a flavonoid), which stimulates mucus secretion and increases mucosal thickness; soluble fibers that buffer gastric acid; and potassium compounds that promote mucosal cell proliferation [31]. Experimental studies have demonstrated its ability to reduce gastric acid secretion and protect against ulcerogenic agents [30,31,32,33]; and (v) keratin is also listed among the formulation ingredients in the original study protocol; its contribution to GERD outcomes was not independently assessed in this survey. Therefore, the synergistic combination of these components simultaneously addresses multiple pathophysiological mechanisms of GERD: the alginate raft mechanically prevents acid reflux; hyaluronic acid and *Althaea officinalis* promote mucosal healing and reduce inflammation; *Musa paradisiaca* controls gastric acidity and stimulates protective mucus secretion. As a result, this multi-target approach may represent a rational and innovative strategy for GERD maintenance therapy.

From a diagnostic perspective, validated self-administered questionnaires have become increasingly important tools in gastroenterological practice, both for initial assessment and for monitoring treatment response. The GERD Impact Scale (GIS) is a validated 9-item questionnaire specifically designed for use in gastroenterological settings, assessing the frequency of typical and atypical GERD symptoms and their impact on daily activities, sleep quality, dietary habits, work performance, and the need for rescue medication [34,35]. The GIS has supporting validation and clinical-practice literature, including an Italian adaptation, and is suitable for structured symptom assessment in routine care [34,35,36]. Consistently, real-world evidence (RWE) studies have gained recognition as a valuable complement to randomized controlled trials (RCTs), particularly in chronic conditions such as GERD, where long-term management, patient adherence, and treatment sequencing in routine clinical practice differ substantially from the controlled conditions of RCTs [37]. Unlike RCTs, which enrol highly selected patient populations under strictly controlled conditions, real-world observational studies capture the full heterogeneity of patients encountered in daily clinical practice, including those with comorbidities, polypharmacy, and variable adherence, potentially improving applicability to routine care, although incomplete follow-up can undermine representativeness. Thus, a real-world experience investigated this multicomponent compound in gastroenterological clinical practice. The primary objective of this multicenter observational study was to describe symptom and safety observations associated with the use of Gastrolaryn^®^ Stick as maintenance therapy following an 8-week course of esomeprazole 40 mg in patients with GERD, assessing whether the symptomatic benefits achieved with PPI therapy are sustained and further consolidated after PPI discontinuation, as measured by the GIS questionnaire.

## 2. Materials and Methods

### 2.1. Study Design

This was a multicenter, prospective, real-world observational study conducted in Italy between May 2025 and December 2025. The study was conducted as a survey (the “GastroLaryn Survey”) involving gastroenterologists operating in outpatient clinical settings across Italy. The present GERD cohort was part of the same overall GastroLaryn observational project on gastric reflux, as the laryngopharyngeal reflux cohort reported separately. Data were collected and analyzed within the common project framework, with separate reporting to address the different clinical populations and specialist assessments. The study collected clinical information in routine outpatient care and involved a commercially available food supplement. The study was not a randomized controlled trial; accordingly, no formal randomization or blinding procedures were applied. No experimental drug or investigational medicinal product was administered; treatments were provided in accordance with routine clinical practice and applicable product instructions. The project was conducted in accordance with the principles of the Declaration of Helsinki. The GastroLaryn observational project was approved by the Institutional Review Board of the Italian Airway Association (AIVAS; protocol 04/24, 4 December 2024). Written informed consent was obtained from all participants before enrolment. The original survey protocol also described a comparator pathway, but the present manuscript reports only the available GERD records from the sequential-management pathway; no analyzable comparator dataset was supplied for this analysis. Thus, this report must be interpreted as an uncontrolled descriptive survey.

### 2.2. Study Population

Seventeen Italian gastroenterologists participated in the study. Patients were eligible for inclusion if they met all of the following criteria: adult patients aged 18 years or older; a previously established diagnosis of erosive or non-erosive GERD; a history of heartburn or regurgitation for ≥3 months prior to study entry; an elevated GIS score at baseline; and willingness to provide informed consent. Exclusion criteria were: Barrett’s esophagus or oesophageal stenosis; active oesophageal, gastric, or duodenal ulcers; history of oesophageal, gastric, or duodenal surgery; active malignancy of any type; pregnancy or breastfeeding; known hypersensitivity to any component of the study product; diagnosis of Helicobacter pylori infection; use of a PPI within 7 days, or any H2-receptor antagonist, prokinetic agent, alginate-based preparation, or antacid within 2 days before screening; and any other condition deemed inappropriate by the investigator. All investigators were required to apply these eligibility criteria.

### 2.3. Treatment Protocol

All enrolled patients received esomeprazole 40 mg once daily for 8 weeks (baseline T0 to T1), in accordance with current guidelines for the management of GERD. Following completion of PPI therapy at T1, patients received Gastrolaryn Stick (1 stick, 15 mL, twice daily after the two main meals) for a further 8 weeks (T1 to T2). The recommended administration of Gastrolaryn Stick was to massage the stick before use, open it, and ingest the contents directly by mouth. This sequential treatment design, such as PPI induction followed by Gastrolaryn Stick maintenance, was chosen to reflect a clinically rational and practically applicable management strategy, evaluating whether the symptomatic benefits achieved with PPI therapy could be sustained and further consolidated after PPI discontinuation.

### 2.4. Assessments and Outcome Measures

Assessments were performed at three timepoints: baseline (T0), after 8 weeks of PPI therapy (T1), and after a further 8 weeks of Gastrolaryn^®^ Stick maintenance (T2).

At each timepoint, the following data were collected:

1. Demographic and anthropometric data (age, sex, BMI).
2. Lifestyle variables (smoking, alcohol consumption, dietary habits, postprandial posture, speed of eating).
3. Concomitant medications.
4. Upper gastrointestinal endoscopy findings documented in routine care and recorded in the survey for the preceding four weeks at each assessment (T0, T1, T2). Repeat EGDS was not imposed by the survey protocol; examinations were performed according to the treating gastroenterologist’s clinical judgement and actual practice.
5. GERD Impact Scale (GIS) questionnaire.

The survey recorded endoscopic findings observed in clinical practice at the three assessment periods. Given its invasive nature, EGDS was not a mandatory study-driven procedure for every patient at every timepoint; the findings reported at each visit correspond to examinations actually performed. Endoscopic information was analyzed descriptively and was not used to infer an intervention-specific mucosal healing effect.

The primary descriptive outcome was the change in GIS response distribution from T0 to T1 and T2. The GIS is a validated 9-item self-administered questionnaire assessing the frequency of: (1) chest pain or pressure; (2) heartburn; (3) regurgitation or acid taste in the mouth; (4) epigastric pain or burning; (5) throat pain or hoarseness related to acid reflux; (6) sleep disturbance due to symptoms; (7) inability to consume preferred foods or beverages; (8) impairment of work performance or daily activities; and (9) use of rescue medication (e.g., antacids). Each item is rated on a 4-point ordinal scale: “every day”, “often”, “sometimes”, and “never” [35]. A higher proportion of “never” responses and a lower proportion of “every day” responses indicate better symptom control.

Secondary outcomes included the endoscopic findings actually recorded during routine clinical care and lifestyle indicators across the three assessments. These data were analyzed descriptively, without attributing changes to one component of the sequential management strategy.

### 2.5. Statistical Analysis

Descriptive statistics summarized available observations at each timepoint. Continuous variables are presented as mean ± standard deviation and range, and categorical variables as frequencies and percentages with the relevant denominators. The GIS results represent the distribution of nine item responses per patient, not independent patient counts. The same clinical cohort was followed over the assessment periods, as confirmed by the investigators. Because the available summary report does not contain the paired, individual-level GIS data needed for within-patient effect estimates, comparisons use descriptive available-case distributions. Missing outcomes were not imputed; a reliable intention-to-treat or multiple-imputation analysis cannot be derived from the aggregate summaries. No inferential or causal treatment-effect analysis was performed.

## 3. Results

### 3.1. Patient Enrolment and Follow-Up

At T0, 263 patients were enrolled by 17 gastroenterologists (mean 15.5 per investigator; range 2–32). Follow-up assessments were available for 124 patients at T1 (47.1% of the baseline cohort) and 112 at T2 (42.6% of baseline), reported by 11 and 7 investigators, respectively. The investigators confirmed that these were follow-up observations in the same original clinical cohort, although the number of available observations varied between visits and centers. In this pragmatic survey, documentation was collected during ordinary outpatient care, without trial-like procedures to enforce return visits or data submission. Accordingly, missing follow-up reflects a genuine challenge of longitudinal monitoring in daily practice. The reasons for missing forms were not systematically collected and cannot be interpreted as evidence of either treatment success or failure. The observed follow-up pattern therefore both illustrates the real-world setting and warrants consideration of attrition bias when interpreting the findings.

### 3.2. Baseline Demographic and Clinical Characteristics

At T0, sex was recorded for 261 of 263 participants: 124 males (47.5%) and 137 females (52.5%); two records lacked sex information. Mean age was 48.4 ± 15.2 years (range 18–87 years). Mean BMI was 25.6 ± 5.2 kg/m^2^ (range 17.2–59; 258 available BMI records). Baseline characteristics are summarized in Table 1.

**Primary descriptive outcome: GIS results**

**Figure 1:**
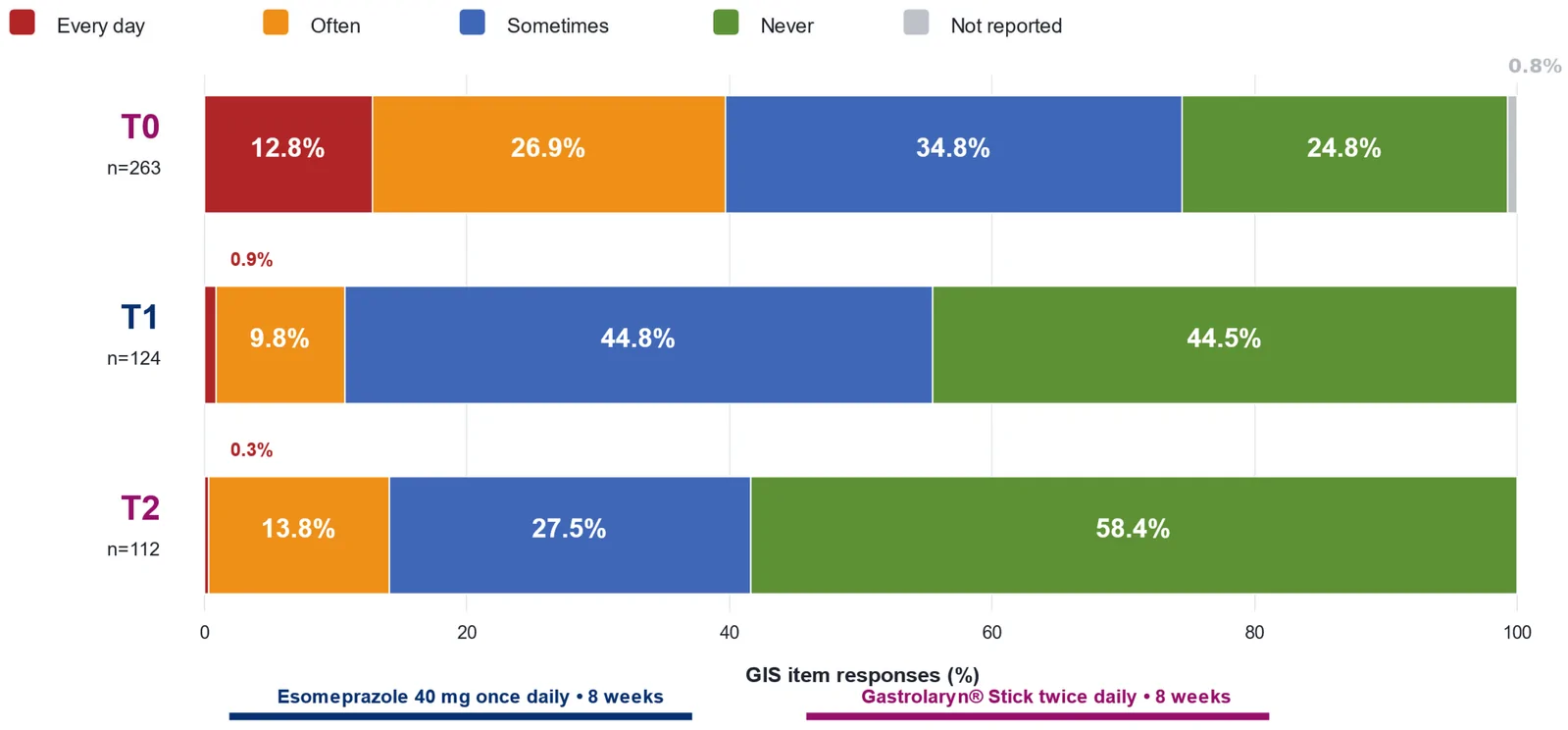
Distribution of GIS response categories recorded at T0, T1 and T2 among patients with available observations at each visit. Results describe the clinical course observed in routine care; denominators vary between assessments.

**Figure 2:**
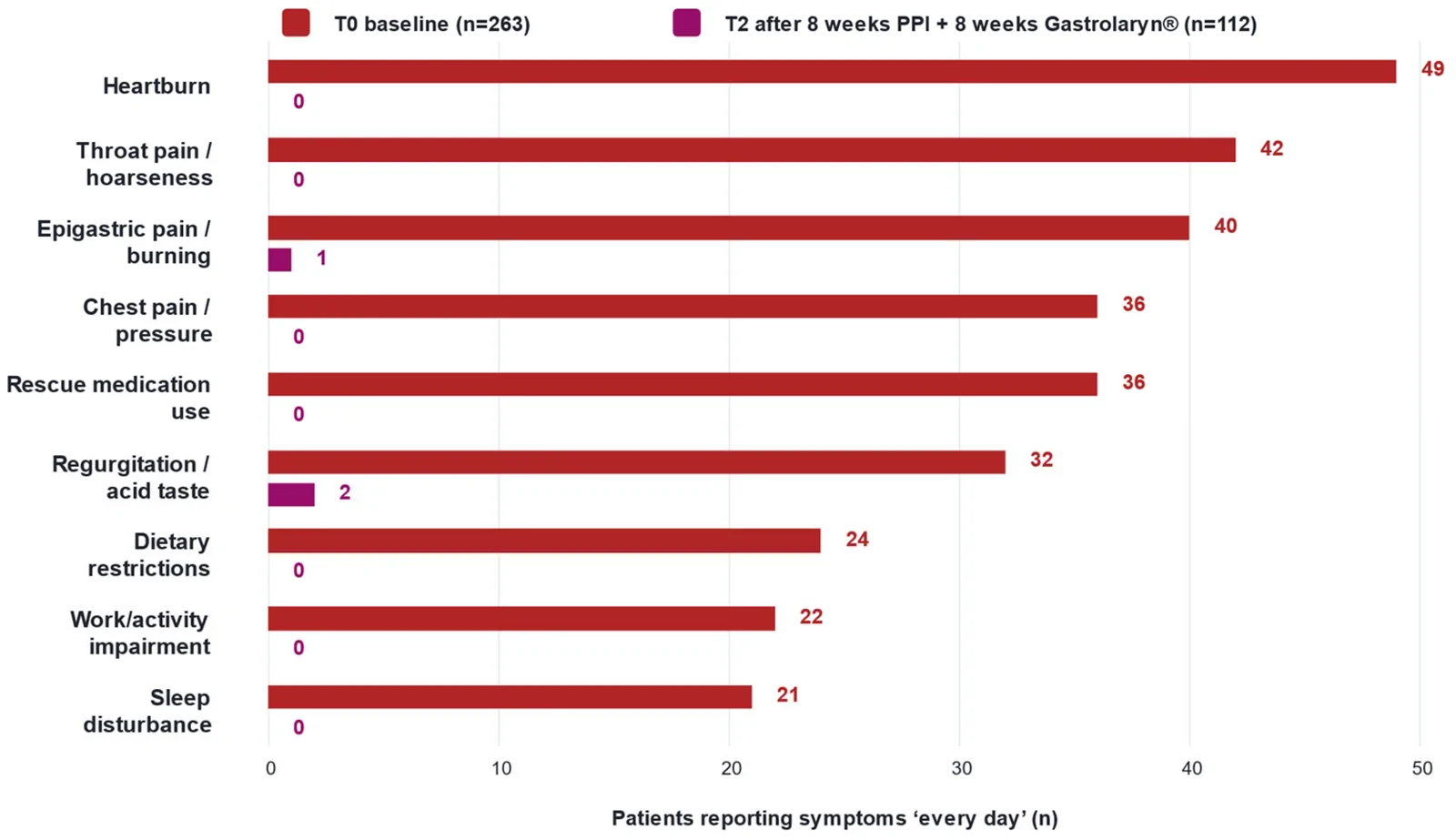
Counts of “every day” GIS responses by item at T0 and T2. Numbers of evaluable observations differed between assessments; results are descriptive.

At T0, the counts of “every day” GIS responses were heartburn (49), reflux-related throat symptoms (42), epigastric pain or burning (40), chest pain or pressure (36), rescue-medication use (36), regurgitation (32), dietary restriction (24), work/activity impairment (22), and disturbed sleep (21). Across 2367 possible GIS item responses (263 × 9), there were 302 “every day”, 637 “often”, 823 “sometimes”, 587 “never”, and 18 missing item responses (0.8% after rounding). These proportions are item-level, not percentages of patients. See Table 2 and Table 3 and Figure 1 and Figure 2.

### 3.3. GIS Results at T1 (After 8 Weeks of Esomeprazole 40 mg)

At T1, 124 sets of GIS responses were reported. Of 1116 item responses, 10 (0.9%) were “every day”, 109 (9.8%) “often”, 500 (44.8%) “sometimes”, and 497 (44.5%) “never”. These distributions differ from the baseline distribution, but the changing composition and substantial reduction in available records mean that individual symptom improvement and an effect of esomeprazole cannot be quantified from these aggregate data.

### 3.4. GIS Results at T2 (After 8 Weeks of Gastrolaryn Stick Maintenance)

**Figure 3:**
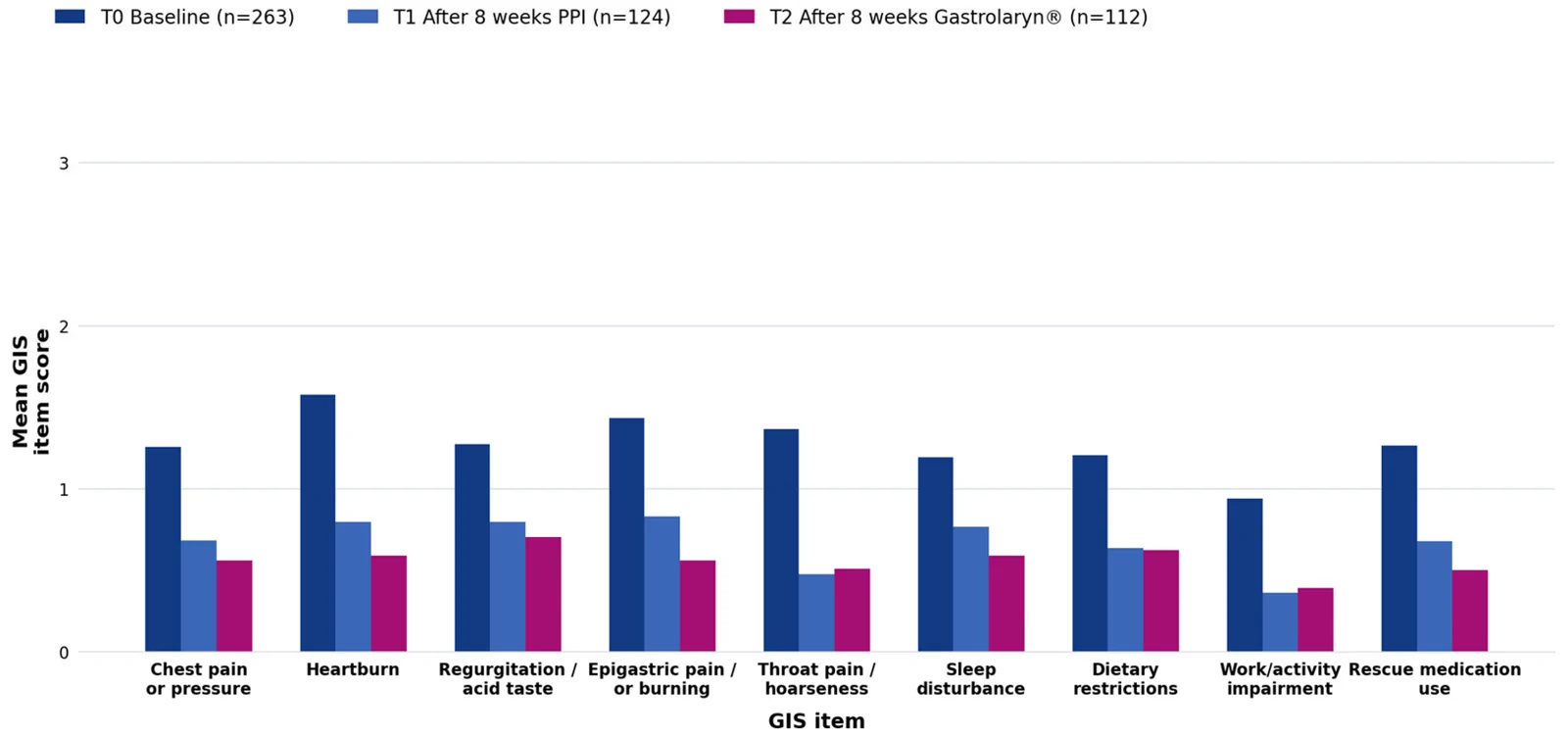
Mean ordinal GIS item scores calculated from available observations at T0, T1 and T2 (0 = never; 3 = every day). Scores reflect observed symptom burden at each assessment.

At T2, GIS observations were available for 112 patients (1008 item responses): 3 “every day” (0.3%), 139 “often” (13.8%), 277 “sometimes” (27.5%), and 589 “never” (58.4%). The corresponding mean GIS item scores across the three assessment timepoints are presented in Figure 3. The three “every day” responses consisted of two for regurgitation and one for epigastric pain. The greater frequency of “never” responses at T2 suggests favourable symptom control among patients assessed in routine care; however, the different denominators and absence of a concurrently analyzed control group prevent an estimate of the product-specific treatment effect.

### 3.5. Secondary Outcome: Lifestyle Variables

At baseline (T0), smoking was reported by 29.3% of patients and regular alcohol consumption by 39.9%. In addition, 52.1% reported eating quickly, 51.7% considered dinner the main meal of the day, and 60.5% maintained correct postprandial posture. Overall, 51.7% reported following a healthy diet. The aggregate lifestyle profile showed that 56% of responses indicated unhealthy behaviours (52% in men and 60% in women). Detailed lifestyle data across all three timepoints are presented in Table 4.

At T1, the proportion of patients following a healthy diet was 79.0%, and the proportion maintaining correct postprandial posture was 60.5%. At T2, 63.4% followed a healthy diet and 48.2% maintained correct postprandial posture.

### 3.6. Secondary Outcome: Upper Gastrointestinal Endoscopy Findings

**Figure 4:**
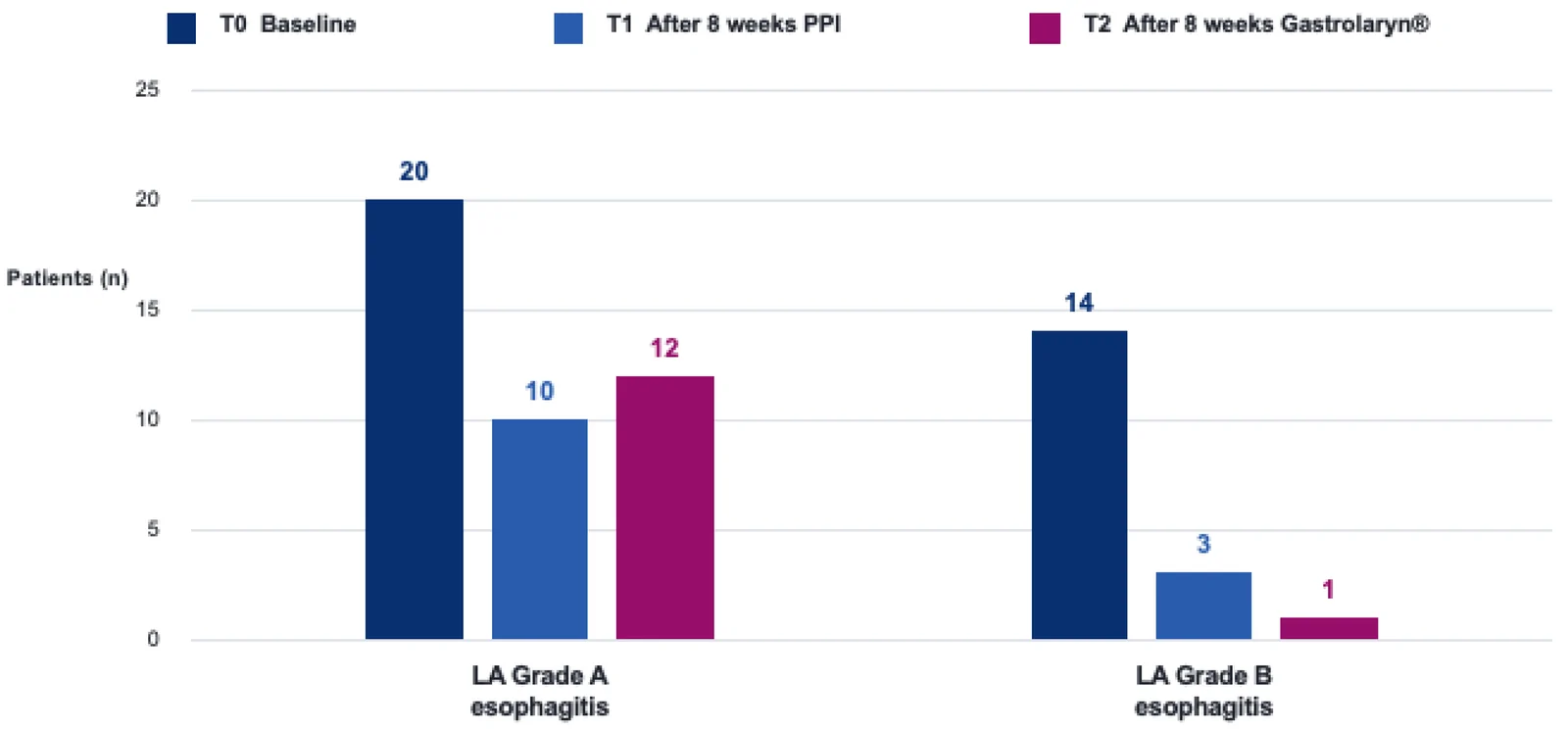
Los Angeles grade A and grade B esophagitis recorded at T0, T1 and T2 during routine clinical care (absolute counts). Because assessments were not available for all patients at every timepoint, these descriptive counts should not be interpreted as paired changes in endoscopic severity.

The recorded upper gastrointestinal endoscopy findings included grade A esophagitis (T0: 20; T1: 10; T2: 12) and grade B esophagitis (T0: 14; T1: 3; T2: 1). The distribution of Los Angeles grade A and grade B esophagitis findings at the three assessment timepoints is illustrated in Figure 4. These values refer to examinations actually reported in routine clinical practice. As the available aggregate report does not provide a matched endoscopic evaluation for every patient at each visit, changes in category frequencies are described without causal attribution.

Endoscopic results reflect actual clinical investigations rather than study-mandated serial examinations. Their pragmatic collection allows an appraisal of findings encountered during usual care, although heterogeneous reporting and variable numbers of examinations restrict direct longitudinal comparisons.

## 4. Discussion

This multicenter observational survey addresses a frequent clinical challenge: how to manage and monitor GERD symptoms after a course of PPI treatment in everyday outpatient practice. The available observations showed a favourable distribution of GIS symptom-frequency responses during the subsequent management period with Gastrolaryn^®^ Stick. The study followed the same original clinical cohort, although not every patient had an available assessment at each visit. Its results therefore describe the observed clinical course under routine-care conditions and provide a practical basis for further investigation of post-PPI symptom management.

### 4.1. Comparison with Existing Evidence

Previous clinical investigations of alginate-based formulations provide a scientific rationale for considering non-PPI approaches during GERD management [38,39,40,41,42,43]. However, differences in product composition, patient selection and study design preclude direct extrapolation of their treatment-effect estimates to Gastrolaryn^®^ Stick. The present observational data add information on the use of a sequential management pathway in everyday care, a context complementary to controlled clinical trials.

### 4.2. Interpretation of GIS Findings

The GIS results document a favourable pattern of symptom-frequency responses across the three survey timepoints. The “every day” category represented 12.8% of all possible item responses at baseline, 0.9% at T1 and 0.3% at T2, while “never” rose from 24.8% to 44.5% and 58.4%, respectively. These findings are of clinical interest because they were observed during routine outpatient management, without the restrictive follow-up procedures of a randomized trial. The number of assessable patients differed between visits, however, and missing outcomes may introduce attrition bias; consequently, the descriptive trends do not by themselves quantify a product-specific causal benefit.

### 4.3. Endoscopic Findings

Endoscopic findings were collected when EGDS had been performed as part of the patient’s ordinary clinical management; no additional invasive examination was required solely for research purposes. This is an important characteristic of the real-world design. The reported distribution of findings at the study visits provides clinical context for symptom outcomes, but cannot establish mucosal healing attributable to the supplement without a standardized matched endoscopy dataset.

### 4.4. Lifestyle Variables

The lifestyle data collected at T0, T1, and T2 provide additional context for interpreting the symptomatic outcomes. At baseline, a high prevalence of modifiable GERD risk factors was documented: 52.1% of patients reported eating quickly, 51.7% considered dinner the main meal of the day, and only 51.7% followed a healthy diet overall. The aggregate lifestyle profile showed that 56% of responses indicated unhealthy behaviors, consistent with the known association between dietary and lifestyle habits and GERD symptom severity [12]. A notable improvement in lifestyle variables was observed at T1, with the proportion of patients following a healthy diet increasing from 51.7% to 79.0%. These improvements were largely maintained at T2 (63.4% healthy diet; 48.2% correct postprandial posture). The concurrent improvement in lifestyle behaviors may have contributed independently to the observed symptomatic improvement. It should be considered in the interpretation of the GIS results, as discussed in the Limitations section.

#### Strengths and Real-World Relevance

A major strength of the GastroLaryn Survey is its close correspondence to everyday gastroenterology practice. It addresses an important and recurring management question—symptom control following an initial PPI course—across multiple outpatient settings, with routine clinical assessments, lifestyle variation and follow-up patterns. This pragmatic framework increases the clinical relevance of the observations and illustrates the feasibility and challenges of collecting longitudinal patient-reported outcomes outside trial conditions. Such practice-based information is valuable when considering the applicability of management strategies in routine care [44].

### 4.5. Clinical Implications

The favourable GIS distributions among patients with available follow-up suggest that sequential management after PPI treatment deserves consideration as a practical research and care question. The GIS offers a simple, patient-centered means of assessing symptom burden over time. Although the study cannot establish the specific treatment effect of Gastrolaryn^®^ Stick, the observations are readily interpretable in the context of outpatient GERD management and may help clinicians identify priorities for structured symptom monitoring and future comparative evaluation.

Prospective comparative studies with more complete longitudinal follow-up, paired symptom data and standardized documentation of concomitant care would be useful to determine whether these favourable symptom patterns persist and to quantify the independent contribution of the supplement.

## 5. Limitations

The principal limitation is incomplete follow-up (124 of 263 patients at T1 and 112 at T2), accompanied by a decrease in the number of reporting centers. Reasons for missing observations were not systematically recorded, and attrition may have affected the representativeness of the available-case symptom distributions. In addition, the available aggregate data do not permit paired patient-level estimates or a defensible missing-data imputation analysis.

Other limitations include the lack of a concurrently analyzed control group, potential confounding by changes in lifestyle and other aspects of routine management, and the selective availability of endoscopic findings. These factors preclude causal attribution of the observed symptom changes to the supplement and limit conclusions about recurrence prevention.

## 6. Conclusions

This multicenter Italian real-world survey addresses a common and clinically relevant challenge: management of GERD symptoms after an initial course of PPI therapy. Among patients with available follow-up, symptom-frequency distributions were favourable during the subsequent period of care with Gastrolaryn^®^ Stick. By documenting a pragmatic sequential pathway and its outcomes under routine outpatient conditions, the study offers practice-relevant evidence that may inform symptom monitoring and the design of future management strategies. While attrition and the absence of a comparator prevent firm conclusions about product-specific efficacy or relapse prevention, the findings support further evaluation of this clinically transferable approach.

## Figures and Tables

**Table 1:** Baseline demographic and clinical characteristics of the study population (T0, *n* = 263).

Characteristic	Value
Total patients enrolled (T0)	263
Male	124 (47.5%)
Female	137 (52.5%)
Mean age (years)	48.4 ± 15.2 (*n* = 263)
Age range (years)	18–87
Mean BMI (kg/m^2^)	25.6 ± 5.2 (*n* = 258)
BMI range (kg/m^2^)	17.2–59
Number of investigators	17
Mean patients per investigator	15.5
Patients at T1	124 records/11 investigators (47.1% of T0)
Patients at T2	112 records/7 investigators (42.6% of T0)
Smokers	77 (29.3%)
Regular alcohol consumption	105 (39.9%)
Eating quickly	137 (52.1%)
Dinner as main meal	136 (51.7%)
Correct postprandial posture	159 (60.5%)
Following a healthy diet	136 (51.7%)
Taking other medications	106 (40.3%)

*BMI: body mass index. Data presented as n (%) or mean ± SD unless otherwise stated.*

**Table 2:** GIS aggregate response distribution at T0, T1, and T2 (% of total item responses).

GIS Response Category	T0 (*n* = 263)	T1 (*n* = 124)	T2 (*n* = 112)
Every day	12.8% (*n* = 302)	0.9% (*n* = 10)	0.3% (*n* = 3)
Often	26.9% (*n* = 637)	9.8% (*n* = 109)	13.8% (*n* = 139)
Sometimes	34.8% (*n* = 823)	44.8% (*n* = 500)	27.5% (*n* = 277)
Never	24.8% (*n* = 587)	44.5% (*n* = 497)	58.4% (*n* = 589)
Not reported	0.8% (*n* = 18)	0.0% (*n* = 0)	0.0% (*n* = 0)
Total responses	2367	1116	1008

Denominators were 2367 possible item responses at T0 (263 × 9), 1116 at T1 (124 × 9), and 1008 at T2 (112 × 9). T0 included 18 unreported GIS item responses. Percentages are recalculated from each timepoint-specific total and may not sum to exactly 100% due to rounding; these are not percentages of participants.

**Table 3:** GIS item-level response frequencies at T0, T1, and T2 (absolute counts).

GIS Item	T0 Every Day	T0 Often	T1 Every day	T1 Often	T2 Every Day	T2 Often
Chest pain or pressure	36	73	1	13	0	17
Heartburn	49	90	1	20	0	19
Regurgitation or acid taste	32	78	1	17	2	16
Epigastric pain or burning	40	80	4	12	1	13
Throat pain/hoarseness (reflux-related)	42	81	0	13	0	18
Sleep disturbance	21	68	2	9	0	17
Dietary restrictions	24	67	1	8	0	12
Work/activity impairment	22	39	0	8	0	18
Rescue medication use	36	61	0	9	0	9

Unpaired counts of “every day” and “often” categories, per GIS item and timepoint; the number of participants differs across timepoints (T0: 263; T1: 124; T2: 112). Counts must not be subtracted across visits to estimate within-patient symptom change.

**Table 4:** Lifestyle variables at T0, T1, and T2 (% of patients responding ‘Yes’).

Lifestyle Variable	T0 (*n* = 263)	T1 (*n* = 124)	T2 (*n* = 112)
Smokers	29.3%	31.5%	26.8%
Regular alcohol consumption	39.9%	21.0%	39.3%
Eating quickly	52.1%	39.5%	53.6%
Dinner as main meal	51.7%	43.5%	58.0%
Correct postprandial posture	60.5%	60.5%	48.2%
Following a healthy diet	51.7%	79.0%	63.4%
Taking other medications	40.3%	37.1%	29.5%

Percentages refer to available timepoint-specific survey samples (T0: *n* = 263; T1: *n* = 124; T2: *n* = 112) rather than a complete set of paired observations. Item-specific nonresponse denominators were not provided; therefore, displayed percentages should be interpreted cautiously.

## Data Availability

The data associated with the paper are available from the corresponding author upon reasonable request.
